# B12 Deficiency-Induced Hemolytic Anemia With an Extensive Deep Vein Thrombosis

**DOI:** 10.7759/cureus.82151

**Published:** 2025-04-12

**Authors:** Bhavya Parikh, Kishan Patel

**Affiliations:** 1 Internal Medicine, Northwell Health, New Hyde Park, USA

**Keywords:** dvt, fatigue, macrocytosis, pernicious anemia, vitamin b12

## Abstract

Vitamin B12 deficiency is a commonly encountered condition in both inpatient and outpatient settings. However, in severe cases, it can have many hematologic complications that can be life-threatening. We present a 56-year-old African American female patient presenting with fatigue found to have a B12 deficiency with elevated methylmalonic levels with simultaneous hemolytic anemia, pancytopenia, and an extensive deep vein thrombosis (DVT) involving her lower extremities. Labs showed anemia, thrombocytopenia, macrocytosis, elevated lactate dehydrogenase (LDH), low haptoglobin, elevated D-dimer, and a positive intrinsic factor blocking antibody leading to a diagnosis of pernicious anemia. Along with anticoagulation, the patient was recommended intramuscular B12 injections daily for seven days followed by weekly and then monthly. Here, we emphasize that B12 deficiency can be life-threatening and requires a high index of suspicion for early recognition of various downstream effects for appropriate management and treatment.

## Introduction

Vitamin B12 (cobalamin) deficiency is a common disorder that is encountered by many clinicians. It is also one of the causes of anemia that comes with its fair share of sequelae. In patients with anemia, 1%-2% of people have a cause of anemia secondary to B12 deficiency [1]. The elderly are more affected, with a lower incidence seen in patients of African descent compared to people of Northern European ancestry [1]. B12 is found largely in animal products, including meat, seafood, fortified cereals, and dairy. It is absorbed in the terminal ileum after binding to intrinsic factors secreted by gastric parietal cells. Deficiency frequently causes megaloblastic anemia characterized by mean corpuscular volume (MCV) > 100. This is a result of ineffective erythropoiesis causing defective DNA synthesis. Cells develop immature nuclei with giant metamyelocytes with macrocytosis and hypersegmented nuclei seen on peripheral smears [2]. Some of the clinical manifestations include neuropsychiatric symptoms such as irritability, depression, paresthesias, weakness, ataxia, decreased reflexes, and sensations secondary to demyelination seen in conditions such as subacute combined degeneration of dorsal column-medial lemniscus and lateral pathways of the spinal cord. Common hematologic conditions include pancytopenia with symptoms attributed to tissue hypoxia. Hypercoagulable states can also be rarely seen in the setting of possible hyperhomocysteinemia [3]. Here, we present a case with a rare manifestation of B12 deficiency highlighted by hemolytic anemia and extensive deep vein thrombosis (DVT) and the importance of recognizing the signs and symptoms. We also highlight the importance of a comprehensive workup to ensure early diagnosis and appropriate management of B12 deficiency.

## Case presentation

A 56-year-old African American female patient presented to the hospital with two weeks of progressively worsening generalized fatigue, lightheadedness, and dyspnea on exertion. The patient had a history of depression and was recently prescribed duloxetine. The patient endorsed some weight loss over the past three months due to decreased PO intake from depression, however, denied any other past medical history. The patient did not follow a vegetarian/vegan diet. She denied any neurological symptoms, including paresthesias, weakness, or sensory changes. She denied any obvious bleeding, extremity pain or swelling, surgery, recent travel, or immobility. The patient was not taking any medications despite being prescribed duloxetine-she had self-discontinued after a week. The patient had a history of five biological children with one miscarriage. She also denied any chest pain, palpitations, orthopnea, or family history of cardiac disease. She endorsed no history of smoking or alcohol use. Moreover, the patient also did not have any history of cancer or lower extremity infection. The physical exam was unremarkable including a full neurological exam with no lower extremity swelling or pain. The patient's BMI was elevated to 34.1 kg/m^2^ consistent with obesity. The patient was noted to be afebrile and normotensive, but tachycardic on vital signs with EKG showing sinus tachycardia without any ST changes.

Lab studies were notable for severe anemia with elevated MCV. The patient was also thrombocytopenic on her complete blood count. On the metabolic panel, the patient was also noted to have an acute kidney injury with serum creatinine elevated and mild elevation in total bilirubin (Table 1).

**Table 1:** Laboratory values with references RBC: red blood cell; MCV: mean corpuscular volume; INR: international normalized ratio; aPTT: activated partial thromboplastin time; LDH: lactate dehydrogenase; TIBC: total iron-binding capacity; TSH: thyroid-stimulating hormone; MMA: methylmalonic acid

Lab	Value	Reference range
Hemoglobin	4.6	11.5-15.5 g/dL
Hematocrit	13.4	34.5%-45%
RBC count	1.16	3.80-5.20 M/μL
Platelets	122	150-400 K/μL
MCV	115.5	80-100 fL
Absolute reticulocytes	13.9	25-125 K/μL
Prothrombin time	12.3	9.5-13.0 sec
INR	1.1	0.85-1.18
aPTT	26.0	24.5-25.6 sec
d-dimer	4,489	<229 ng/mL
Fibrinogen	212	200-465 mg/dL
LDH	4,306	135-225 U/L
Haptoglobin	<20	34-200 mg/dL
Total bilirubin	1.8	0.2-1.2 mg/dL
Creatinine	2.79	0.5-1.30 mg/dL
Ferritin	437	13-330 ng/mL
TIBC	193	220-430 μg/dL
% iron saturation	42%	14%-50%
Vitamin B12	150	200-900 pg/mL
TSH	1.34	0.27-4.20 μIU/mL
Folate	15.6	3.1-17.5 ng/mL
MMA	94,746	0-378 nmol/L
Anticardiolipin antibody	Negative	Negative
Intrinsic factor blocking antibody	29.3	0-1.1 AU/mL
Direct Coombs IgG & C3	Negative	Negative
ADAMTS 13	Negative	Negative

Further workup was obtained showing decreased B12 levels with normal folate levels. This prompted further evaluation showing significantly elevated methylmalonic acid (MMA) levels with a peripheral smear demonstrating granulocytes with hypersegmentation. Moreover, an intrinsic factor blocking antibody was also significantly elevated.

**Figure 1:**
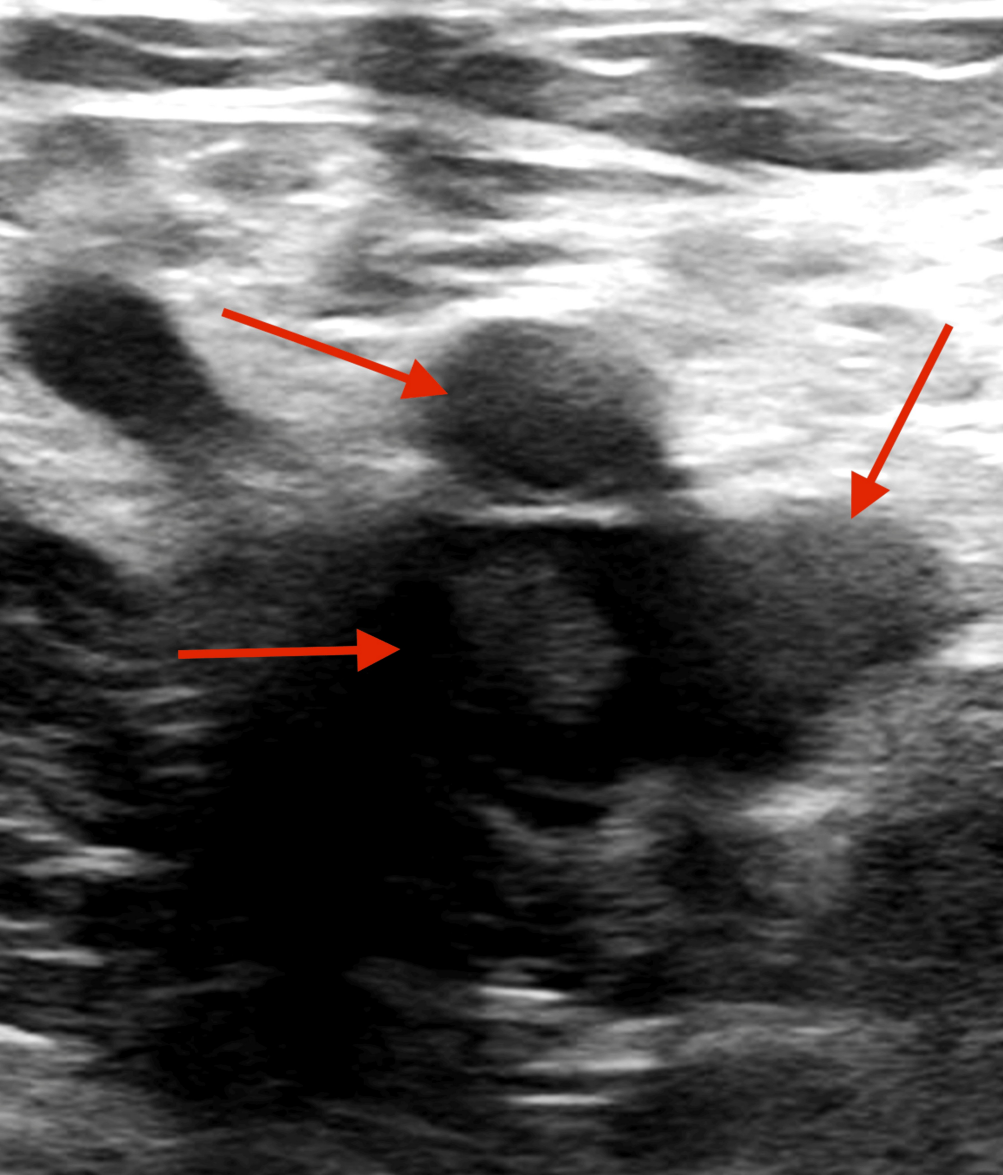
DVT observed during vascular studies DVT: deep vein thrombosis

Hemolysis studies showed a severely elevated lactate dehydrogenase (LDH) with an unmeasurable haptoglobin of <20. The patient also had a significantly elevated d-dimer. A duplex study of the lower extremity showed an extensive DVT above and below the knee, including external iliac, common femoral, greater saphenous, and popliteal veins in the left extremity (Figure 1). Here, since multiple deep veins were affected, a thrombectomy was planned with the vascular surgery team.

The patient, in the meantime, was supportively transfused and placed on full anticoagulation with unfractionated heparin in light of the extensive DVT. The patient was also prescribed intramuscular (IM) B12 1,000 mcg injections daily for seven days, then once weekly after. The patient displayed hemodynamic stability during her stay with no signs of hypoxia, worsening tachycardia, and/or hypotension. No new neurological abnormalities were observed during her stay. The patient was discharged prior to thrombophilia genetic testing, but it was recommended to be obtained as an outpatient. Stress testing could also be indicated for further coronary artery testing.

## Discussion

**Figure 2:**
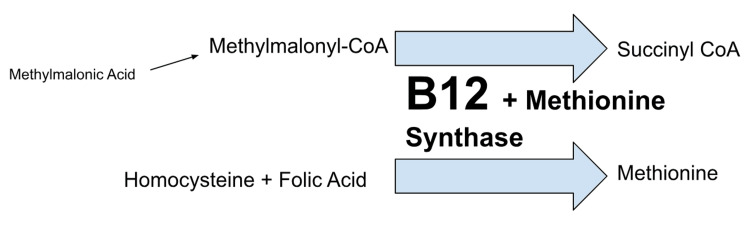
Role of B12 in metabolizing MMA and homocysteine B12 deficiency build-up leads to increased MMA and homocysteine levels MMA: methylmalonic acid

Here, we present a patient with a rare manifestation of B12 deficiency with pernicious anemia and concurrent hemolysis, with pancytopenia, and extensive thrombosis. There have been some prior studies noting the correlation between elevated risk of thrombosis and true B12 deficiency [3]. Specifically, in cases of hyperhomocysteinemia, prior data has shown elevated risks for strokes, myocardial infarctions, and atypical thromboses. The best data comes from a case-control study done by den Heijer et al., which established high plasma homocysteine levels as a risk factor for thrombosis in 269 patients [4]. However, a direct link with vitamin deficiency was not explored. As per Figure 2, biochemically, vitamin B12 is a co-factor for methionine synthase, which helps in the catalyzation of homocysteine to methionine and 5-methyltetrahydrofolate to tetrahydrofolate, which is in turn used for proper DNA synthesis. Elevated homocysteine levels are seen in both deficiencies in the clinical presentation of this patient, as folate levels were normal. Here, elevated MMA levels can differentiate between a folate deficiency and a B12 deficiency as the true cause.

There have been many theories presented in the literature as to what causes the elevated risk of venous thrombosis and atherosclerosis in B12 deficiency. Specifically, hyperhomocysteinemia seen in B12 deficiency has been shown to interfere with vascular endothelial function with the induction of tissue factor activity or protein C activation [5]. Specific molecular mechanisms underlying prothrombosis are incompletely understood, but they involve oxidative stress, DNA hypomethylation, and proinflammatory effects. For this patient, although non-specific, d-dimer elevation was helpful in suspecting a DVT in the absence of any prior history of cancer or acute infection, while the homocysteine and MMA levels were pending. This prompted a vascular study of lower extremities, which was swiftly obtained and markedly affected inpatient management.

Furthermore, concurrent hemolysis was evidenced by elevated LDH levels with severely low haptoglobin levels and elevation in total bilirubin. Our patient did not have any history of anemia in the past nor did she require transfusion; there was no evidence of hepatosplenomegaly and no abdominal tenderness; hence, an intra-abdominal pathology/inherited condition was lower on the differential. Since the Coombs test, cold agglutinin test, and APLS labs were also negative, it argued against an autoimmune process. Recently started outpatient duloxetine has rarely been seen as a cause of DVT in literature and was held inpatient. Microangiopathic causes such as thrombocytopenic purpura (TTP), hemolytic uremic syndrome (HUS), and disseminated intravascular coagulation (DIC) were also less likely due to the negative ADAMTS 13, acute kidney injury (AKI) normalization upon transfusion, and coagulation factors (prothrombin time (PT)/international normalized ratio (INR) and activated partial thromboplastin time (aPTT)) being normal upon arrival. This is an important set of differentials to consider, as the management can be varied for each. In the literature, many cases of vitamin B12 deficiency have been shown to cause microangiopathic anemia, leading to a false TTP diagnosis and the initiation of plasmapheresis [6].

Theoretically, hemolysis seen in B12 deficiency is thought to be due to elevated homocysteine levels, which in vitro studies have shown to be due to possible lipid peroxidation and cytoskeletal lipid derangement [7]. Homocysteine has also been noted as a potential “hemolytic toxin” due to the pro-oxidant effect that stems from the generation of free radicals during the oxidation of varied mixed disulfide compounds [8].

It is quite rare for dietary B12 deficiency to manifest such acutely with concurrent hemolysis and thrombosis as it takes about 5-10 years in Western nations due to large stores in the liver. Typically, pernicious anemia is responsible for about 75% of cases of B12 deficiency and its clinical sequelae, as seen in our patient. It is typically more common in the elderly population due to gradual degradation and atrophy of the gastric mucosa leading to malabsorption [9].

The patient here was recommended IM B12 injections daily for seven days, followed by weekly injections for four weeks, and then monthly. The patient’s hemolysis markers were trended daily while inpatient as a timeline for improvement in B12 deficiency. Specifically, improvement in reticulocytosis, decreasing LDH levels, and an appropriate rise in hemoglobin levels can be trended while receiving treatment. Appropriate outpatient follow-up is necessary regarding continuing full anticoagulation and B12 therapy. These markers can be tested by the primary care physician (PCP) prior to seeing a hematologist outpatient as well. Anticoagulation duration is often an individualized decision based on the risk of recurrence, genetic testing results, and the resolution of B12 deficiency.

## Conclusions

B12 deficiency commonly presents with neuropsychiatric disturbances and hematological abnormalities. This case represents a complex presentation of B12 deficiency with hemolysis and pancytopenia. We highlight the important aspects of the differential diagnosis and workup that should be considered in such a presentation and the importance of ruling out TTP, HUS, and DIC and evaluating for downstream effects like venous thromboembolisms (VTEs) in the setting of elevated homocysteine/MMA levels. Intrinsic factor antibodies are also important to obtain if suspecting pernicious anemia. The diagnosis can be confirmed with B12 supplementation and reduction in LDH levels, improved reticulocytes, and rise in hemoglobin levels. Future studies are needed to explore the incidence of thrombosis and hemolysis in pernicious anemia as well. Thus, B12 deficiency can have a complex presentation that can be easily missed causing a host of down-the-line life-threatening complications and comorbidities if not addressed promptly.
